# A knowledge-based approach to designing control strategies for agricultural pests

**DOI:** 10.1016/j.agsy.2020.102865

**Published:** 2020-08

**Authors:** Annika Agatz, Roman Ashauer, Paul Sweeney, Colin D. Brown

**Affiliations:** a Department of Environment and Geography, University of York, Wentworth Way, Heslington, York, YO10 5NG, United Kingdom; b Syngenta, Jealott's Hill, Maidenhead Rd., Warfield, Bracknell, RG42 6ES, United Kingdom

**Keywords:** Corn rootworm, Maize, Integrated pest management, Insecticide, Individual based model, Yield loss

## Abstract

Chemical control of insect pests remains vital to agricultural productivity, but limited mechanistic understanding of the interactions between crop, pest and chemical control agent have restricted our capacity to respond to challenges such as the emergence of resistance and demands for tighter environmental regulation. Formulating effective control strategies that integrate chemical and non-chemical management for soil-dwelling pests is particularly problematic owing to the complexity of the soil-root-pest system and the variability that occurs between sites and between seasons. Here, we present a new concept, termed COMPASS, that integrates ecological knowledge on pest development and behaviour together with crop physiology and mechanistic understanding of chemical distribution and toxic action within the rhizosphere. The concept is tested using a two-dimensional systems model (COMPASS-Rootworm) that simulates root damage in maize from the corn rootworm *Diabrotica* spp. We evaluate COMPASS-Rootworm using 119 field trials that investigated the efficacy of insecticidal products and placement strategies at four sites in the USA over a period of ten years. Simulated root damage is consistent with measurements for 109 field trials. Moreover, we disentangle factors influencing root damage and pest control, including pest pressure, weather, insecticide distribution, and temporality between the emergence of crop roots and pests. The model can inform integrated pest management, optimize pest control strategies to reduce environmental burdens from pesticides, and improve the efficiency of insecticide development.

## Highlights

• New systems-based model for root damage/yield loss due to corn rootworm in maize.
• 92% predictive accuracy for 119 field trials covering four sites and ten seasons.
• Optimisation of design and use of insecticides can reduce environmental burdens.
• Key role for lag between root emergence and egg hatch in integrated pest management.

## Graphical abstract

**Figure:**
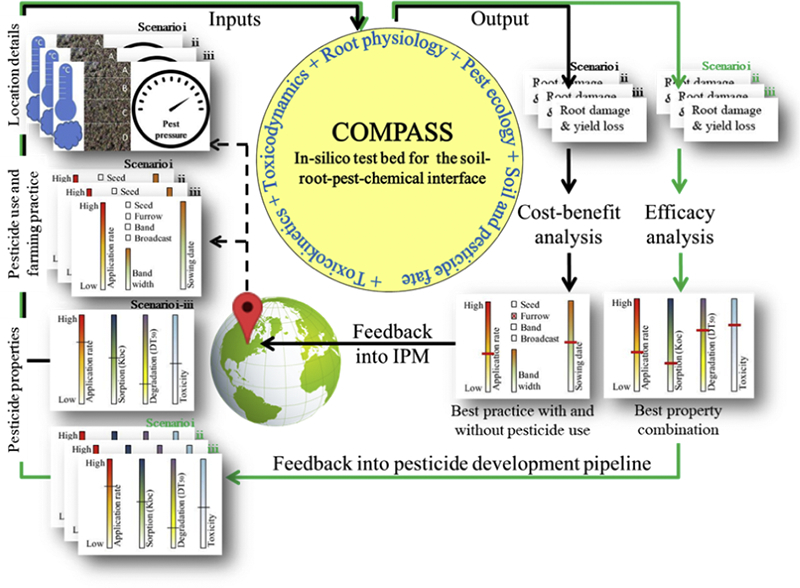

## 1 Introduction

Global crop production depends on efficient protection from insect damage. Wheat, rice, maize, barley, potato, soybean, sugar beet, and cotton yield losses attributed to animal pests (primarily insects) were estimated to be 10% in 2002, ranging from 7% in barley to 24% in rice and 37% in cotton; losses to animal pests constituted 58% of the theoretical loss in the absence of crop protection measures (Oerke, 2006). Modern crop protection from insect pests is delivered through a combination of breeding and varietal selection, rotation, soil and crop husbandry, biological and microbial control, and chemical insecticides.

In particular, chemical insecticides have been a cornerstone of agricultural intensification since the introduction of organochlorine insecticides in the 1960s (Peshin et al., 2009). Gianessi (2009) estimated that US growers spent $1.2 billion on insecticides in 2008 to treat 17% of the 1.1 million km^2^ of land cultivated with the 50 main crops, and that this resulted in a yield benefit of $22.9 billion. More than 650 insecticides have been registered for use on the global market. Compared with the earliest insecticides, modern active substances have improved mammalian toxicological profiles and greater selectivity; targeted placement into the crop, for example as a seed treatment or banded application, can achieve use rates lower than 50 g of active substance (a.s.)/ha (Lamberth et al., 2013).

Despite these advances, there are major challenges to the continued availability of insecticides, owing to the combined effects of pest resistance, changing pest distributions, regulatory pressure on existing products, and inefficiencies in the development of new products. Globally, nearly 600 species of insects have been reported to be resistant to one or more of 325 insecticides and/or five genetically modified insecticidal traits (Sparks and Nauen, 2015); this necessitates the development, maintenance, and use of pesticides with a variety of modes of action, and the adoption of integrated pest management to minimize chemical interventions (Wilson et al., 2018). Trade and transport have been important factors in the spread of pest species, and climate change will modify the range of agricultural pests. Substantial positive latitudinal shifts in pest populations have been observed in the Northern Hemisphere from 1960 onwards, with Acari, Coleoptera, Hemiptera, Lepidoptera, and Fungi shifting polewards and Nematoda shifting toward the Equator (Bebber et al., 2013).

Environmental concerns about the use of insecticides have resulted in tighter regulatory control. Stehle and Schulz (2015) found that 52% of 11,300 reported concentrations of insecticides in surface waters exceeded regulatory threshold levels where effects on macroinvertebrates can be expected. Exposure to pollen and nectar contaminated with neonicotinoid insecticides has been identified as one of several factors implicated in the global decline in the number of pollinators (Sanchez-Bayo and Goka, 2014). Furthermore, insecticide use on crops has been linked to declines in terrestrial biodiversity in general (Geiger et al., 2010) as well as to declines specifically in the number of farmland birds (Mineau and Whiteside, 2013). Regulatory schemes have responded to these findings with high-profile actions, such as the moratoriums imposed by many countries on neonicotinoid seed treatments, and through restrictions and deregistration of active substances already on the market (Balderacchi and Trevisan, 2010).

Agrochemical discovery is almost exclusively based on a “chemistry-first” paradigm that has focused on the identification of leads through high-throughput, in vivo testing of chemical libraries (Lamberth et al., 2013). Mean times to discovery have remained in the range of 3–4 years for the past half-century, with a 70-fold increase in the number of compounds screened per product discovered (currently ca. 160,000) counteracted by the adoption of virtual screening and the use of techniques from pharmaceutical discovery, such as structure-based design, fragment-based design, and genome sequencing (Lamberth et al., 2013). Post-discovery, the cost (up to $286 million) and time (8–12 years) required to take a new pesticide through the development and registration process have increased significantly (Sparks and Lorsbach, 2016). These increases have led to a reluctance to invest in pest control for minor use crops (Sparks and Nauen, 2015).

Product development accounts for ca. 50% of the total cost to introduce a new agrochemical (Sparks and Lorsbach, 2016), and comprises a linear sequence of steps, from high-throughput identification of an efficacious lead using standard bioassays, to laboratory studies on target pests, glasshouse experiments, and finally field studies using a range of crops and environmental conditions (Kalamarakis and Markellou, 2007). The development sequence is largely empirical, with little feedback of mechanistic information into upstream development processes.

This study argues for a new approach to developing pest control strategies that is termed COMPASS (Comprehensive Model for Pesticide Activity in Soils). The concept integrates mechanistic understanding of pest ecology, insecticide fate, and insecticide effects on the pest into a systems-based, spatially-explicit model of root damage by pests. The models that result from taking such an approach deliver an in silico testbed of the soil-root-pest-chemical interfaces acting within the soil profile that aims to capture variability in influencing factors across different sites and agricultural seasons.

We illustrate the COMPASS concept with the COMPASS-Rootworm model using a test system comprising corn (*Zea mays* L.) root damage and yield losses caused by the corn rootworm (*Diabrotica* spp.). Corn rootworm is a commercially significant pest that is widespread in North America, Central America, and Europe, with annual yield and control losses in the USA alone that were estimated to be greater than $1 billion between 2005 and 2007 (Dun et al., 2010). We demonstrate the validity of the COMPASS approach using a decade of field data (119 field trials) for the chemical control of corn root damage by rootworm larvae (University of Illinois Extension, 2005–2014). We quantify the benefits in terms of agricultural yields, environmental protection, and integrated pest management, and discuss step changes to our understanding of crop protection that can be delivered through the application of this knowledge-based approach to the design of pest control strategies.

## 2 Materials and methods

### 2.1 Field dataset

A dataset of 122 field efficacy trials was compiled and split into subsets comprising three trials used for model parameterization and 119 independent trials used for model evaluation. The trials collectively applied 11 plant protection products containing the active substances clothianidin (a neonicotinoid, CAS 210880–92-5), chlorpyrifos (an organophosphate, CAS 2921–88-2), or tefluthrin (a pyrethroid, CAS 79538–32-2) as seed, furrow, or band applications over a period of ten years (2005–2014) at four trial sites in Illinois, USA: Urbana (40.07, −88.21), Perry (39.79, −90.82), DeKalb (41.84, −88.86), and Monmouth (40.93, −90.72) (University of Illinois Extension, 2005–2014). Supplementary Data (SD) Table S1 provides an overview of the field trials, whilst SD Table S2 gives details of the design for each trial. Planting time across the 122 trials ranged from Julian day 107 to 143 (April 16th to June 12th).

Each trial comprised four replicate plots for a given treatment and the control. Damage assessment was undertaken 62 to 111 days after planting by extracting and washing five root systems per replicate plot and then using the Node Injury Scale (NIS; Oleson et al., 2005) to quantify damage, with values ranging from 0.00 (no damage) to 3.00 (three full nodes or the equivalent across all nodes are lost; the maximum possible value).

### 2.2 COMPASS-Rootworm model

**Fig. 1:**
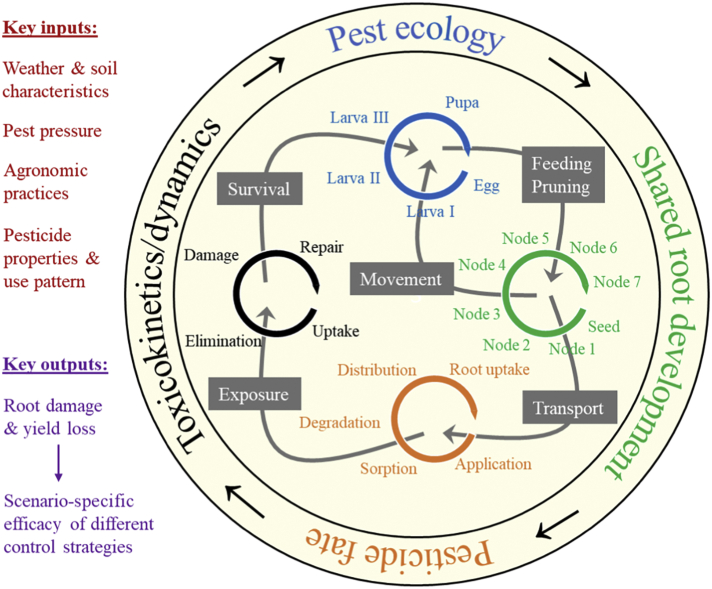
Integration of spatially and temporally explicit models within the COMPASS-Rootworm modeling framework showing the primary processes (**outer circle and text in inner circle**) and model connections (**arrows in inner circle**). Key model inputs and outputs are provided to the left.

COMPASS-Rootworm is a two-dimensional systems model that describes the development and spatial distribution of corn roots and corn rootworm pest, as well as the fate, distribution, and toxic action of chemical insecticide; in combination, this allows simulation of root damage (and hence yield loss) by the pest under different environmental conditions, as well as the effectiveness of different chemical and non-chemical control strategies. The primary processes, inputs and outputs for the model are illustrated in Fig. 1.

COMPASS-Rootworm is coded in the freely available NetLogo 5.3 (Wilensky, 1999). The spatial discretization describes a vertical soil profile of 76 × 100 cm (x- and y-axes) and 1 cm depth (z-axis) that was selected to simulate a cross-section through the root system of a single corn plant in a field with row spacing of 76 cm. Each patch/grid represents 1 cm^3^. Most processes are updated once per hour, and all the processes are updated at least once a day. The model runs from the beginning of the calendar year to the day of the NIS assessment using Julian days as the temporal measure. The model runs start at the beginning of a year, as both the pest model and the pesticide fate model require site-specific weather data preceding the actual corn and corn rootworm season to simulate the temporal and two-dimensional occurrence and distribution of the root, pest, and pesticide owing to their dependency on temperature and water content in the soil profile.

### 2.3 Pest ecology

Pest ecology is described with an individual-based population model for the western corn rootworm *Diabrotica virgifera virgifera* (discretized at a 1-cm resolution) (Agatz et al., 2017). A spatially-explicit root growth submodel describes the stochastic appearance and development of new roots within a defined nodal structure. The pest model simulates the development of eggs, the three larval instars, and the pupa of the corn rootworm, with the transition between life stages controlled by temperature-dependent developmental-rate functions. Speed of larval movement toward food (and, thus, larval foraging success and the survival of both larvae and roots) is dependent on soil type (Strnad and Dunn, 1990). The larval instars feed at different rates and consume roots of different ages (Clark et al., 2006; Agatz et al., 2017). Root consumption is linked to root damage (NIS) and yield loss through the direct loss of biomass and pruning of roots. Site-specific weather data (air temperature and precipitation) are used to generate two-dimensional profiles of soil temperature and water content, which in turn drive the dynamics between pest and root systems. A previous evaluation using long-term data for Central Illinois showed that the corn rootworm model predicted the date of the first appearance of both larvae and adults to within one week of the first recorded sightings and accurately simulated the site- and season-specific root damage recorded in the field in the absence of pest control (Agatz et al., 2017).

Initial pest pressure (i.e., the density of eggs in the soil from the preceding season) is an important input to the model, but is not normally measured in field studies of corn rootworm. The previous evaluation of the pest model demonstrated that measured damage is simulated accurately for control plots when pest pressure is measured and used directly as a model input (Agatz et al., 2017), so we conclude that pest pressure can be parameterized. Thus, we used measured damage in the control plots of the field trials to parameterize pest pressure (a single value for all trials undertaken at each unique site-year combination; SD Table S2) and then held this value constant in model runs to simulate the impact of the various pesticide treatments on root damage. As all sites were ploughed, egg positioning was set to the tillage option (Agatz et al., 2017) with homogeneous distribution in the horizontal plane, and vertical distribution as given by Vidal et al. (2005) (21% of eggs within the upper 10 cm, 45% of eggs at 10–20 cm, and 34% of eggs at 20–30 cm).

### 2.4 Pesticide fate

The model of pesticide fate in soil simulates temporally and spatially explicit water and pesticide transport in the soil profile by running simulations from Julian day 1 (January 1st) until harvest under the influence of the explicit and stochastic development of root segments on a daily basis (Agatz and Brown, 2017). This procedure allows the model to describe the microscale movement of pesticides in relation to root segments, which represents an important addition relative to existing models of pesticide fate in soil. In addition to the root growth model described above, the crop processes within the model include shoot development, spatially explicit uptake of water in response to transpiration demand, interception of rainfall and irrigation by the canopy, and evaporation from the canopy. The model captures spatial variation in inputs of precipitation (due to canopy interception and stem flow) and irrigation to the soil surface. Movement of water within the soil profile is modeled using the soil capacity approach and occurs sequentially in the horizontal and vertical planes; the rate of water movement is defined using a maximum hydraulic gradient that is user-defined in each plane. Currently the model is set up for flat sites, but it could be modified to incorporate the effect of a sloping site on water transport. Uptake of water by roots occurs locally in soil according to the spatial distribution of root segments. Upward movement of water can occur in response to evaporation from the soil surface and root uptake, but water that flows from the base of the soil profile is considered lost from the system.

Pesticides can be applied to the soil as a seed treatment or as an in-furrow, banded, or broadcast application, and they are subsequently subject to first-order degradation and linear, instantaneous sorption, with both processes modified according to the water content and temperature of the respective 1-cm^3^ grid. Dissolved pesticides are available for transport through soil with moving water and uptake by roots adjusted for the relative ease of uptake of different pesticides (Agatz and Brown, 2017).

### 2.5 Toxicity to rootworms

Toxic action of insecticide on rootworms is described using the toxicokinetic-toxicodynamic approach of the General Unified Threshold Model of Survival (GUTS; Jager et al., 2011). Rootworm larvae experience variable pesticide concentrations in the soil within the model owing to their own movement and the fate of the compound in the soil profile. GUTS translates the time-variable pesticide concentration in soil experienced by each individual larva into a predicted probability of survival. GUTS-RED-SD (i.e. the reduced version of GUTS following the stochastic death assumption for survival; Jager and Ashauer, 2018) was parameterized using ModelMaker (v.4.0, AP Benson, Wallingford, UK) and acute toxicity experiments reported by Agatz et al. (2018). Larvae of the rootworm were exposed to different concentrations of one of the three chemicals homogeneously mixed into silt loam soil, and the number of surviving larvae was recorded after 24, 72, and 120 h (SD Table S3). Calibrated parameter values are shown in Table 1.

**Table 1:** Compound-specific parameter values for the COMPASS-Rootworm model. Three parameters of the GUTS-RED-SD special case model (Jager et al., 2011; Jager and Ashauer, 2018) are used to characterize lethal effects, and the parameter *Prun-red* is used to account for sublethal effects.

Parameter	Description (unit)	Compound (life stage)	Value
*k*_d_	Dominant rate constant (d^−1^)	Clothianidin (L2) Chlorpyrifos (L2) Tefluthrin (L2)	100 100 4.374
*b*_w_	Killing rate constant (L/ mg d^−1^)	Clothianidin (L2) Chlorpyrifos (L2) Tefluthrin (L2)	3.693 0.887 1.497
*z*_w_	Threshold (mg/ L)	Clothianidin (L2) Chlorpyrifos (L2) Tefluthrin (L2)	0.032 0.035 0
*Prun-red*	Relative pruning reduction accounting for sublethal impacts on reduced feeding damage (−)	Clothianidin (L2) Chlorpyrifos (L2) Tefluthrin (L2)	0.62 0.954 1.00

Previous rhizotron experiments carried out with field-relevant application rates and placement strategies demonstrated that clothianidin, chlorpyrifos, and tefluthrin can induce reduced feeding in rootworms, either by direct feeding inhibition or by impairing their ability to sense or move toward food (Agatz et al., 2018). In those experiments, clothianidin had a repellent effect on larvae, whereas chlorpyrifos provoked premature pupation and reduced the growth of larvae in comparison to nonexposed organisms. Tefluthrin caused a loss of control over movement. At present, we lack the toxicokinetic-toxicodynamic models for the sublethal effects of chemical toxicants in soil pest species that would have allowed the simulation of the impacts on larval feeding (Ashauer and Jager, 2018). Instead, we incorporated a compound-specific parameter (*prun-red*) into our modeling framework that accounts for the sublethal impacts of a compound in terms of a reduction of root-pruning damage relative to that in the absence of the compound. For each compound, this parameter was fitted to NIS data from one field trial carried out in Urbana in 2007 (i.e. the training set comprised three of the 122 field trials; Table 1). The calibrated value of *prun-red* for each compound was then held constant in all simulations of the remaining 119 independent field trials used for model evaluation.

### 2.6 Model evaluation

Field trials in the evaluation set were modeled according to the information obtained for the field trial site (application time, application rate, application type, duration for the trial, i.e. sowing time to day of harvest; SD Table S2) using weather data (obtained from the Illinois Climate Network 2016) recorded by weather stations in Bondville, DeKalb, Monmouth, and Perry that are located close to the University of Illinois field stations where the trials were undertaken. The USDA (2017) Web Soil Survey tool was used to generate site-specific soil information for the simulations (SD Table S4). The physicochemical properties of the active substances used were derived from the literature (University of Hertfordshire, 2013) and are summarized in SD Table S5. We considered that a predicted NIS damage value was in agreement with the observed NIS damage value if the 95% confidence interval of the predicted NIS damage value fell within ±0.83 NIS damage units of the observed NIS damage value, as explained in the results section.

### 2.7 Limiting factors for pesticide efficacy

To analyze the interplay between the NIS and the application rate, we used the weather profile recorded for Urbana (IL, USA) in 2014 and ran the model framework by applying increasing application rates of clothianidin at planting on Julian day 131 (May 10th) as a furrow application on Drummer soil (SD Table S4), with efficacy assessment 72 days after planting. The initial pest pressure was 108 eggs/L soil.

To analyze the interplay between placement strategy and application rate, we simulated the efficacy of clothianidin in one region and one season by altering the compound application type and rate using the weather profile recorded for Urbana (IL, USA) in 2006, while keeping the planting day as Julian day 131, with efficacy assessment 72 days after planting, pest pressure of 45 eggs/L soil, and the Drummer soil type constant. Model predictions were analyzed in SigmaPlot (version 12.5, Systat Software, San Jose, CA, USA) using a global exponential rise to maximum (3 parameters) fit to the average of all the model predictions (*N* = 40).

The effects of the physicochemical properties of a hypothetical chemical compound on pest control were investigated by simulating the scenario-specific efficacy of a compound (GUTS parameters: dominant rate constant, *k*_d_ = 1000 1/d, killing rate constant, *b*_w_ = 0.378 L/mg/d, threshold, *z*_w_ = 0.112 mg/L; parameter to account for sublethal impacts, *prun-red* = 0) for three seasons (2005, 2011, and 2014) at one location while varying Koc and DT50 from starting values of 56 mL/g and 121 days, respectively. Scenario specifications were Drummer soil with a seed application rate of 0.6 mg a.s./seed; weather profiles for Urbana (IL, USA) from 2005, 2011, and 2014; pest pressures for 2005, 2011, and 2014 of 161, 91, and 108 eggs/L soil, respectively; planting on Julian day 122 (May 1st), 130 (May 10th), and 131 (May 11th), respectively; and efficacy assessment 71, 61, and 72 days after planting, respectively (see SD Table S2 for details).

A cost-benefit analysis was undertaken for several simulations. Details are provided in the SD.

### 2.8 Integrated pest management

Globally, there is demand to adopt agronomic practices with reduced pesticide inputs. For example, the EU Sustainable Use Directive defines integrated pest management as a system that integrates measures to discourage the development of populations of harmful organisms, considers all available plant protection methods, and only uses control methods to levels that are economically and ecologically justified (Lefebvre et al., 2015). Hatching of pest larvae is determined by soil temperature (degree days above a threshold; Davis et al., 1996) and extent of root damage in the absence of control measures is strongly influenced by the relative timing of emergence of roots and pest larvae. The model framework captures this effect of inter-year variability in weather conditions, so it could be used to deliver reduced pesticide usage by identifying those conditions where insecticide use is not economically and ecologically justified, and/or by optimizing sowing date in a given year to reduce the need for pest control. This was investigated for all data summarized in SD Table S2 by normalizing the observed NIS damage values in the control plots and the simulated NIS damage values from all treatment plots to a single, common pest pressure of 45 eggs/L soil and normalizing the temporal variation in the data by correlating the normalized NIS damage values to the number of days between the simulated root emergence and the day the first egg developed into a larvae.

## 3 Results and discussion

### 3.1 COMPASS-Rootworm performance

The efficacy to control root damage by corn rootworm (indicated by a reduction in NIS) of products tested in the 119 field trials used for model evaluation ranged from 0 to 99% (median 76%); the value for each trial was calculated from averages given in reports, and assuming 0% efficacy for trials where the damage to plants in treated plots was equal to or greater than that in the untreated controls. The amount of rootworm damage in the control plots varied among trial sites and years, with the greatest overall damage sustained in Urbana and the least overall damage sustained in Perry. SD Fig. S1 illustrates the variability in the efficacy of products tested in each field trial by plotting the NIS damage values recorded in the treatment plots against those observed in the control plots. In 6.6% of the trials used for evaluation, the average NIS damage value in the control plot was lower than in the treatment plot, with a maximum discrepancy of 0.83 NIS damage units. Owing to the absence of a direct measure of uncertainty associated with the NIS assessments (because raw data for the field trials were not accessible), we used this difference of ±0.83 NIS damage units as an indication of the overall uncertainty of the results from the field trials. SD Fig. S1 shows that in 38 of the 119 trials, the difference in the NIS damage value between the treatment and control was less than 0.83, indicating that in 32% of all trials, the efficacy of the pesticide to reduce pest pressure was not confirmed. Oleson et al. (2005) note that precision in node injury assessment is a function of both extent of root damage and sample size.

**Fig. 2:**
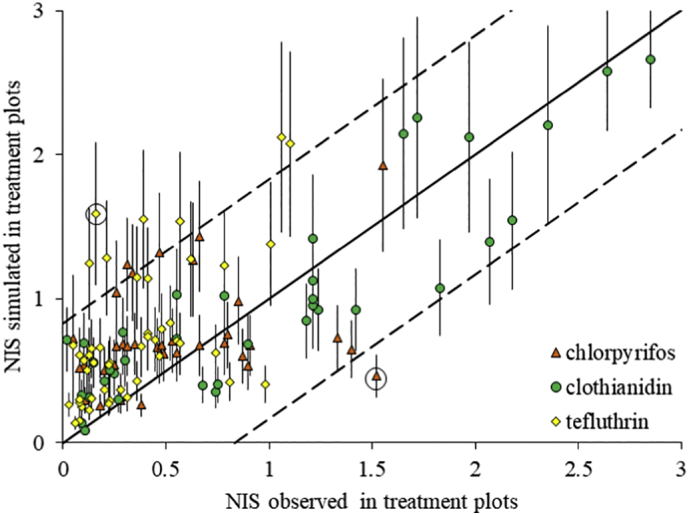
Simulated vs. observed damage (given according to the node injury scale (NIS)) for 119 field trials conducted in Illinois with plant protection products containing chlorpyrifos, clothianidin, or tefluthrin. Simulated results were obtained using the COMPASS-Rootworm modeling framework. The **solid line** represents the situation where NIS is identical for simulations and observations in treated plots and the **dashed lines** show the overall uncertainty around the solid line that is associated with the field trials (±0.83 NIS). The **error bars** indicate the 95% confidence interval of the simulations (*N* = 40). **Circled points** indicate the trials in which the 95% confidence interval of the simulations and the maximum error from field trials do not overlap (2 out of 119 field trials).

COMPASS-Rootworm was able to simulate the outcome of the 119 field trials with all three active substances investigated and across the large range of observed efficacy. Overall, the average of simulated root damage (*N* = 40) was within ±0.83 NIS of the average value observed in the field for 91% of trials (95% confidence interval 80–98%; Fig. 2, SD Table S2). Thus, the model framework accounts for the combined effects of environmental, chemical, and biological factors that determine the efficacy of a plant protection product for a given site/season combination within our dataset. This variation in environmental conditions between sites and years has been identified previously as an important source of unexplained variability across large databases of field efficacy trials (Tinsley et al., 2016). Future work should expand the evaluation to a wider set of conditions, particularly for locations impacted by corn rootworm in Central America and Europe. Rasche and Taylor (2019) also demonstrated successful simulation of field efficacy trials by coupling a crop model with an above-ground insect population dynamics model. There, the authors found that they had to calibrate insecticide dose-mortality relationships to account for laboratory to field extrapolation. In contrast, the current study simulates toxicity mechanistically based on toxicokinetics/toxicodynamics allowing a successful simulation of field effects using parameters derived from laboratory toxicity tests.

The simulation performance tended to decrease with increased strength of pesticide sorption to soil (Fig. 2); 100% of the simulations were within the uncertainty of the field trials for the most weakly sorbed compound, clothianidin, and this decreased to 89 and 85% of the simulations for the more strongly sorbed compounds chlorpyrifos and tefluthrin, respectively. The soil-water partition coefficient which defines sorption is a sensitive parameter in all pesticide fate models (Dubus et al., 2003) and stronger sorption will act to decrease the volume of the root zone where the pesticide is present at any given point in time. When separating the field trials into those that demonstrated the efficacy of a product (i.e., the difference between control and treatment NIS damage values was greater than the uncertainty of the field trials) and those that did not meet this criterion, then the model was able to predict 86% of the 81 field observations proving efficacy and 95% of the 38 field observations that did not show efficacy. Variability in product performance across a limited number of field trials (Toth et al., 2020) can be a major constraint on product development. A field study may fail to demonstrate efficacy for an otherwise efficacious product because of low pest pressure (Furlan et al., 2006), a redistribution of the pesticide within the soil profile that occurs too quickly or too slowly depending on rainfall (Sutter et al., 1989; Sutter et al., 1991), the use of an application strategy that limits efficacy (Tinsley et al., 2016), or because too much time elapses between pesticide application and the appearance of the pest (Sutter et al., 1989). A mechanistic model that can be used as a virtual field study to explain apparent anomalies in product performance will thus strengthen product development.

### 3.2 Limiting factors for pesticide efficac**y**

**Fig. 3:**
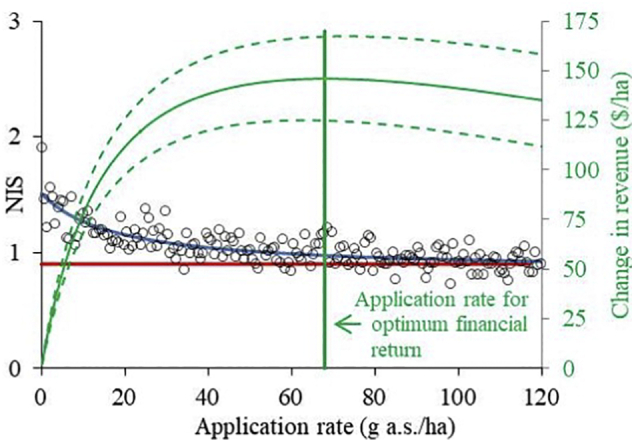
Damage and revenue curve for clothianidin used as furrow treatment in one region and one season as a function of the application rate. **Circles**: Simulated damage (given according to the node injury scale (NIS)) as a function of the application rate as average of 40 COMPASS-Rootworm simulations for each different application rate (**blue line**: fit through data; R^2^ = 0.976). The **red line** indicates the limit to product efficacy for this scenario. The **green curve** (with the associated standard deviation (**dashed curves**)) illustrates the scenario-specific pesticide-related revenue as a function of the application rate. The **vertical green line** indicates the application rate where revenue in relation to pesticide cost is at its maximum. (For interpretation of the references to colour in this figure legend, the reader is referred to the web version of this article.)

Field efficacy trials serve to optimize the application parameters for the product (Kalamarakis and Markellou, 2007). We used COMPASS-Rootworm to evaluate root damage for one environmental scenario as a function of the application rate of clothianidin applied as a furrow treatment at the time of sowing (Fig. 3). The model demonstrates that the insecticide cannot deliver full control of root damage in this scenario; thus NIS decreased as the amount of insecticide applied to the system increased, but only up to a limit value of 0.95 NIS (dotted red line in Fig. 3). The model simulations indicate that compound distribution within the soil profile was the limiting factor preventing further control because the compound did not reach all parts of the soil profile where the pest causes damage to the root system. COMPASS-Rootworm allows calculation of the application rate that provides an optimal economic return (68 g a.s./ha) based on the costs of pesticide application versus yield lost to the pest (Fig. 3), and thus supports the aim of sustainable intensive agriculture by delivering a secure supply of food while minimizing the use of agrochemicals (Popp et al., 2013). The model shows that for this scenario, the farmer could reduce the application rate by 49% from the economic optimum to 35 g a.s./ha and still deliver 92% of the maximum pest control and 98% of the maximum financial return.

**Fig. 4:**
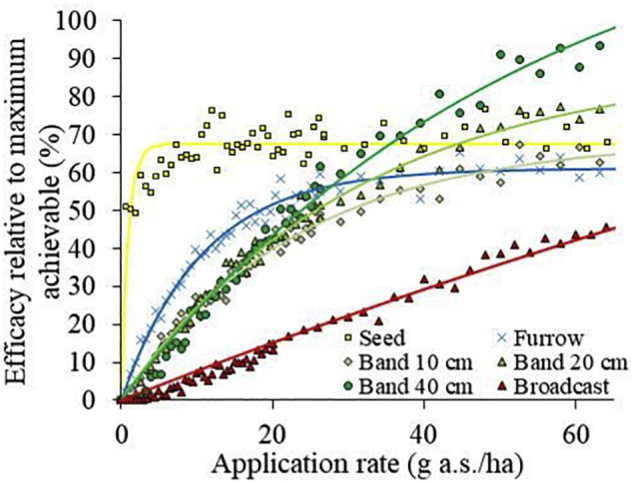
COMPASS-Rootworm simulated scenario-specific efficacy of clothianidin in one region and one season relative to the maximum efficacy that can be delivered at an assumed maximum application rate of 65 g a.s./ha (i.e. band application with 40 cm band width). Data are shown for seed, furrow, broadcast and three band applications of different band width (10, 20, and 40 cm) as a function of the application rate. **Lines**: global fit to the average of all the model predictions (*N* = 40 per application rate) with a 3 parameter exponential rise to maximum (R^2^ = 0.983).

One response to the residual root damage that is demonstrated in Fig. 3 could be to use a modified formulation or product placement strategy for clothianidin (Buntin and All, 2013). Fig. 4 presents a virtual field trial where COMPASS-Rootworm was used to assess four different product placement strategies for one season/soil combination: seed treatment, and furrow, band, and broadcast application. Seed treatment was the most effective strategy at low application rates, delivering 70% of the maximum achievable efficacy at 10 g a.s./ha. At higher application rates, the 40-cm band application was most efficacious, surpassing seed treatment at a rate of 35 g a.s./ha. This is in line with Tinsley et al. (2016) who constructed efficacy functions using corn rootworm control trials from Illinois and Nebraska, concluding that seed treatments were unlikely to be as effective as soil insecticide treatments (at full dose, seed treatments resulted in an average 86% greater damage), Here, narrower band applications and furrow application were not optimal strategies for the scenario evaluated. In practice, broadcast application would have provided the highest level of control of all, but this would have required application rates two- or tenfold greater than those for banded and seed treatment applications, respectively (data not shown). A detailed regional analysis of placement strategies, application rates, and treatment costs with COMPASS could produce farm decision trees to balance the competing demands of high financial return and low risk to the environment. Rossi et al. (2019) identify calibration and validation of decision tools as a critical factor for successful uptake by end users.

**Fig. 5:**
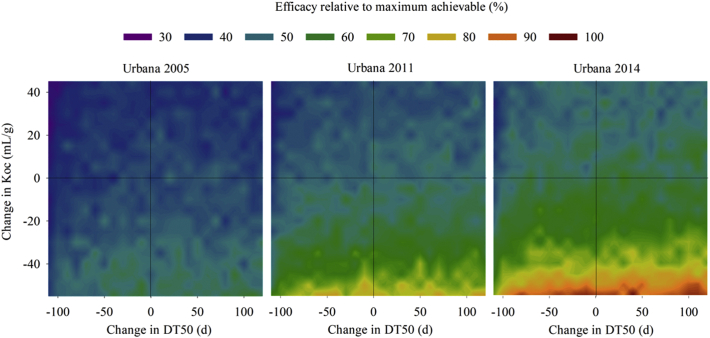
COMPASS-Rootworm simulated efficacy relative to the maximum achievable efficacy of a hypothetical active substance applied as seed treatment over three growing seasons (labels at top) in response to varying physicochemical properties of the compound (Koc: organic carbon partition coefficient, DT50: half-life). The **black lines** indicate the Koc (56 mL/g) and DT50 (121 d) of the pesticide simulated as the base-case.

The development of a class of insecticides generally begins with discovery of a new structural class, followed by modifications of functional groups around the central scaffold (Lamberth, 2018). COMPASS can be used to inform this development by simulating changes to the physicochemical properties of a pesticide that will modify its fate in soil and, thus, modify interactions with the target pest. We defined a hypothetical insecticide and used COMPASS-Rootworm to investigate how pest control would change in response to changes in the mobility and persistence of the compound, expressed as the organic carbon partition coefficient (Koc) and degradation half-life (DT50), respectively. The analysis considered three seasons with contrasting weather conditions and pest pressures. We found that mobility had a much stronger influence on efficacy than persistence with a decrease in Koc of ca. 40 mL/g consistently doubling the efficacy of the hypothetical compound, even though there was a large variation in the absolute efficacy values across the three seasons (Fig. 5). The model results indicate that the spatial co-occurrence of pest larvae and pesticides was a more important limitation to efficacy than the period over which the pesticide was biologically active in soil. If mechanistic information relating to the optimal physicochemical properties were to be fed back into the selection of agrochemical leads (Rao et al., 2015) and product development pipeline, this would reduce the cost and time currently required to take a new pesticide through to registration.

### 3.3 Integrated pest management

**Fig. 6:**
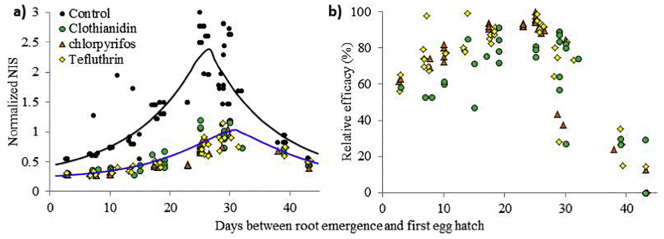
COMPASS-Rootworm simulated average damage and pesticide efficacy plotted against the interval between root emergence and the first egg hatch. **a)** Rootworm damage shown as the node injury scale (NIS). **b)** Pesticide efficacy relative to the damage in the corresponding control. Data have been normalized to a consistent pest pressure of 45 eggs/L soil: trials with NIS in the control plot <0.20 and > 2.80 are excluded owing to uncertainty in assigning the level of root damage at very small or large values for NIS. **Lines**: global fit to all model predictions (*N* = 40) with a 3 parameter exponential rise to maximum (R^2^ = 0.803).

Hatching of pest larvae is determined by soil temperature (degree days above a threshold; Davis et al., 1996), and the relative timing of emergence of roots and pest larvae determines the extent of root damage in a particular year and in turn will strongly influence the efficacy of insecticide use (Clark et al., 2006). However, this effect is masked in the field dataset because pest pressure also varies between sites and seasons (5–164 eggs/L soil in our dataset), and this has a strong influence on root damage and insecticide efficacy. To overcome this problem, we used COMPASS-Rootworm to reanalyze the complete field dataset for insecticide efficacy with the effect of pest pressure on root damage eliminated by normalizing the field trials to a consistent pest pressure of 45 eggs/L soil. Fig. 6a (black dots/line) shows that when larvae emerged very soon (<10 days) after first root emergence, pest damage was minimal because the spatial extent of roots was limited, meaning that few larvae intercepted roots before they died from starvation. Normalized root damage increased exponentially up to a peak in NIS of 2.27 at an interval of 26 days between root emergence and egg hatch. At longer intervals, root damage decreased, because the roots were older at onset of pest pressure, and older roots are known to be unpalatable to first instar larvae^24^. Although all pesticide treatments with clothianidin, chlorpyrifos, and tefluthrin reduced root damage relative to the control (Fig. 6a; colored dots/blue line) the reduction in the normalized NIS compared to the control was only above the uncertainty for NIS of 0.83 when egg hatch occurred 19–29 days after root emergence. Fig. 6b shows that the relative efficacy of all three compounds increased as the time interval between root emergence and egg hatch increased, and was maximal when egg hatch occurred 20–30 days after root emergence. At short intervals between root emergence and egg hatch, our model indicates that the spatial redistribution of a pesticide within the soil profile limited the zone providing effective protection to the growing roots. After approximately 30 days, there was a very sharp decrease in the relative pesticide efficacy and our model suggests that this is because the root system outgrew the soil zone with efficacious concentrations of pesticide (typically for corn root nodes 4 and above). Malard et al. (2020) also identified a strong influence of timing on the success of pest control action when modeling the lepidopteran *Opisina arenosella* as a pest of coconut farming in Sri Lanka; use of their modeling approach to determine optimal timing of biological control was found to outperform timing that was either on a fixed date or determined by pest population thresholds.

Given that egg hatch is determined by soil temperature, and thus independent of root emergence, our analysis identifies a strategy for integrated pest management because sowing at or just before the time of egg hatch carries a low risk of root damage. Specifically, when the interval between root emergence and egg hatch is less than 10 days, the NIS is generally less than 0.5 NIS; this value was calculated as the threshold below which pesticide application is not economic assuming treatment costs of $36/ha (Alford and Krupke, 2017). Medium-range weather forecasting is increasingly reliable out to 10 days; this allows prediction of site-specific timing of egg hatch (Agatz et al., 2017), and supports decisions regarding the optimal time for sowing and either a reduced pesticide application intensity such as in-furrow treatment, or potentially the omission of pesticide treatment altogether. By reducing both the frequency and intensity of insecticide use, this approach could contribute to controlling the development of pest resistance to chemical insecticides (Sparks and Nauen, 2015). Where plowing is planned, site-specific prediction of egg hatch also offers the potential to bring forward egg hatch by plowing; this would expose eggs (which are most abundant at 11–20 cm depth; Vidal et al., 2005) to the warmer upper soil layers thus encouraging earlier egg hatch, and the effect can be simulated in the model as it accounts for redistribution of eggs due to plowing. The alternative strategy of aiming to maximize the time between root emergence and egg hatch is not plausible because this would require accurate long-range forecasting of at least 30 days.

## 4 Conclusion

Insect pests are a global threat to agricultural productivity and this threat is increasing due to changing pest distributions and spread of resistance to chemical insecticides, at the same time as the public is demanding more sustainable agricultural systems. Despite the high level of threat, development of pest control strategies ultimately depends on field studies that are largely empirical. The COMPASS-rootworm model delivers a knowledge-based approach to the design of control strategies for a globally-significant pest that integrates knowledge across the disciplines of pest ecology, root physiology, soil hydrology, and insecticide fate and toxicity. Evaluation of the model against an extensive dataset of field trials for root damage in maize caused by the corn rootworm shows that the model is able to capture much of the variability in damage to maize crops from corn rootworm and effectiveness of pest control strategies that is seen across different locations and agricultural seasons. The approach that is presented delivers virtual field trials, allowing the development of mechanistic understanding of the system. This allows, for example, the optimization of insecticide selection and use to achieve maximum efficacy with minimal risk to the environment or normalization of yield loss to different pest pressures, weather, or treatment timing to develop guidance on integrated pest management strategies and resistance management. Whilst the research presented here is framed by agricultural losses due to corn rootworm, the new approach will have applications across a much wider range of agricultural pests.

## Appendix A Supplementary data

Supplementary material Image 1
