# Supplementary material for: A knowledge-based approach to designing control strategies for agricultural pests
Source: Agric Syst. 2020 Aug;183:102865. doi: 10.1016/j.agsy.2020.102865 (PMC7294735; doi:10.1016/j.agsy.2020.102865)
Supplement: Supplementary file 1 — Supplementary material [file mmc1.docx]

**A knowledge-based approach to designing control strategies for agricultural pests**

Annika Agatz^a,^^[[1]](#footnote-1)^, Roman Ashauer^a,^^[[2]](#footnote-2)^, Paul Sweeney^b^, Colin D. Brown^a,*^

^a^ Department of Environment and Geography, University of York, Wentworth Way, Heslington, York, YO10 5NG, United Kingdom

# ^b^ Syngenta, Jealott's Hill, Maidenhead Rd., Warfield, Bracknell, RG42 6ES, United Kingdom

**^*^** Colin Brown; Department of Environment & Geography, University of York, Wentworth Way, Heslington, York, YO10 5NG, United Kingdom; [colin.brown@york.ac.uk](mailto:colin.brown@york.ac.uk); Tel: 00 44 1904 324322

Supplementary Data


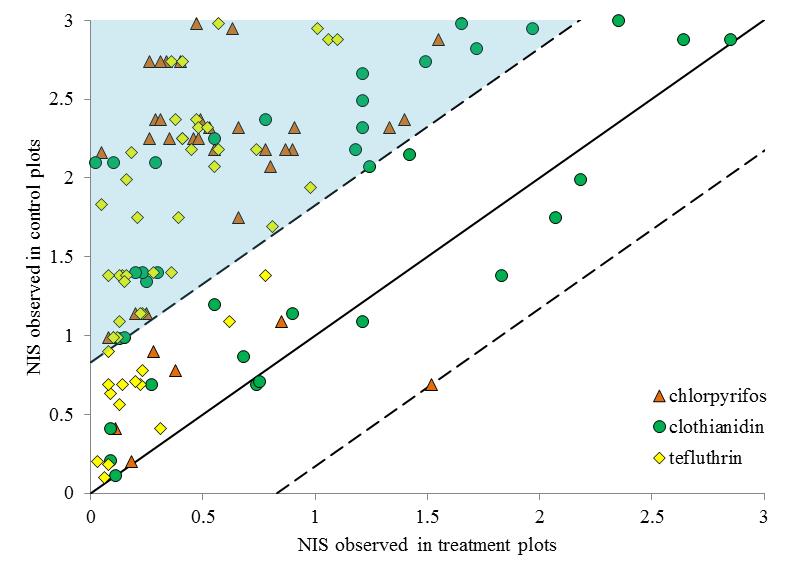


Figure S1: Summary of field trials used to evaluate COMPASS-Rootworm. The average node injury scale (NIS) values observed in control plots compared to those in treatment plots are shown. The colored area illustrates those trials where the efficacy of the treatment against the western corn rootworm was proved. The solid line represents the situation where NIS is identical for control and treated plots and the dashed lines show the overall uncertainty around the solid line that is associated with the field trials (±0.83 NIS).

Table S1: Summary of field trials used to evaluate COMPASS-Rootworm. Shown is the total number of field trials for a given substance-product-application combination and the variation in trial locations and seasons for each combination.

| Active substance | Product[^1^](#_ENREF_1) | Application types[^1^](#_ENREF_1) | Application rate (mg a.s./m of row or mg/seed for seed treatment) | Number of trials | Number of trial sites | Number of years tested |
| --- | --- | --- | --- | --- | --- | --- |
| Chlorpyrifos | Lorsban 15G | Band | 111.61 | 19 | 4 | 5 |
|  | Lorsban 4E | Band | 111.39 | 7 | 4 | 2 |
|  | Lorsban 75 WG | Band | 83.71 | 3 | 3 | 1 |
|  | Nufos 15G | Band | 111.61 | 3 | 3 | 1 |
|  | Saurus 15G | Band | 111.61 | 2 | 2 | 1 |
|  | Saurus 15G | Furrow | 111.61 | 2 | 2 | 1 |
| Clothianidin | Belay liquid | Furrow | 7.70 | 2 | 2 | 1 |
|  | Belay liquid | Furrow | 11.98 | 2 | 2 | 1 |
|  | Belay liquid | Furrow | 13.26 | 4 | 3 | 2 |
|  | Poncho 1250 | Seed | 0.6 | 29 | 4 | 5 |
| Tefluthrin | Force 2.1CS | Band | 10.01 | 6 | 4 | 2 |
|  | Force 2.25CS | Band | 2.61 | 1 | 1 | 1 |
|  | Force 2.25CS | Furrow | 2.61 | 1 | 1 | 1 |
|  | Force 2.25CS | Band | 10.01 | 12 | 4 | 2 |
|  | Force 3G | Band | 11.16 | 14 | 4 | 4 |
|  | Force 3G | Furrow | 11.16 | 1 | 1 | 1 |
|  | Force 3G | Band | 11.16 | 3 | 3 | 1 |
|  | Force CS | Band | 10.01 | 8 | 4 | 2 |
|  | Force CS | Furrow | 10.01 | 3 | 3 | 1 |

Table S2: Summary of field trials from the literature [1] used for COMPASS-Rootworm evaluation and simulation results for the fitted pest pressure and the predicted damage. Observed and simulated damage is given according to the node injury scale [2]. All trials were conducted with a seeding rate of 13,355 seeds ha^–1^ and a row spacing of 76 cm.

| No. | Location  ^*^ | Planting date  ^*^ | Assessment date  ^*^ | Treatment  ^*^ | % active ingredient in product | Pesticide application (oz/1000 ft of row for band and furrow application; mg/seed for seed application) ^*^ | Application rate (mg a.s. /m row) or mg/seed for seed treatment | Active substance | Placement type  ^*^ | Injury value observed in the control  ^*^ | Fitted pest pressure for the control | Injury value observed in the treatment^*^ | Injury value predicted average | Injury value predicted upper 95% CI | Injury value predicted lower 95% CI |
| --- | --- | --- | --- | --- | --- | --- | --- | --- | --- | --- | --- | --- | --- | --- | --- |
| 1 | DeKalb, IL | 27/04/2005 | 26/07/2005 | Lorsban 15G | 15 | 8 | 111.61 | Chlorpyrifos | b | 2.37 | 95 | 0.29 | 0.68 | 0.89 | 0.47 |
| 2 | DeKalb, IL | 27/04/2005 | 26/07/2005 | Lorsban 4E | 49.9 | 2.4 | 111.39 | Chlorpyrifos | b | 2.37 | 95 | 0.49 | 0.62 | 0.81 | 0.43 |
| 3 | DeKalb, IL | 27/04/2005 | 26/07/2005 | Lorsban 75 WG | 75 | 1.2 | 83.71 | Chlorpyrifos | b | 2.37 | 95 | 1.40 | 0.65 | 0.85 | 0.45 |
| 4 | DeKalb, IL | 27/04/2005 | 26/07/2005 | Nufos 15G | 15 | 8 | 111.61 | Chlorpyrifos | b | 2.37 | 95 | 0.31 | 0.67 | 0.88 | 0.46 |
| 5 | DeKalb, IL | 27/04/2005 | 26/07/2005 | Poncho 1250 | 48 | 1.25 | 0.6 | Clothianidin | s | 2.37 | 95 | 0.78 | 1.02 | 1.34 | 0.70 |
| 6 | DeKalb, IL | 27/04/2005 | 26/07/2005 | Force 3G | 3 | 4 | 11.16 | Tefluthrin | b | 2.37 | 95 | 0.47 | 0.60 | 0.79 | 0.42 |
| 7 | DeKalb, IL | 27/04/2005 | 26/07/2005 | Force 3G, modified SmartBox metering units | 3 | 4 | 11.16 | Tefluthrin | b | 2.37 | 95 | 0.38 | 0.67 | 0.87 | 0.46 |
| 8 | DeKalb, IL | 27/04/2006 | 25/07/2006 | Lorsban 15G | 15 | 8 | 111.61 | Chlorpyrifos | b | 2.07 | 79 | 0.80 | 0.75 | 0.98 | 0.52 |
| 9 | DeKalb, IL | 27/04/2006 | 25/07/2006 | Poncho 1250 | 48 | 1.25 | 0.6 | Clothianidin | s | 2.07 | 79 | 1.24 | 0.92 | 1.21 | 0.64 |
| 10 | DeKalb, IL | 27/04/2006 | 25/07/2006 | Force 3G | 3 | 4 | 11.16 | Tefluthrin | b | 2.07 | 79 | 0.55 | 0.71 | 0.93 | 0.49 |
| 11 | DeKalb, IL | 27/04/2006 | 08/08/2006 | Poncho 1250 | 48 | 1.25 | 0.6 | Clothianidin | s | 2.15 | 79 | 1.42 | 0.92 | 1.21 | 0.64 |
| 12 | DeKalb, IL | 03/05/2007 | 16/07/2007 | Lorsban 15G | 15 | 8 | 111.61 | Chlorpyrifos | b | 2.18 | 79 | 0.90 | 0.53 | 0.70 | 0.37 |
| 13 | DeKalb, IL | 03/05/2007 | 16/07/2007 | Lorsban 4E | 49.9 | 2.4 | 111.39 | Chlorpyrifos | b | 2.18 | 79 | 0.55 | 0.62 | 0.82 | 0.43 |
| 14 | DeKalb, IL | 03/05/2007 | 16/07/2007 | Saurus 15G | 15 | 8 | 111.61 | Chlorpyrifos | b | 2.18 | 79 | 0.87 | 0.60 | 0.78 | 0.41 |
| 15 | DeKalb, IL | 03/05/2007 | 16/07/2007 | Saurus 15G | 15 | 8 | 111.61 | Chlorpyrifos | f | 2.18 | 79 | 0.78 | 0.69 | 0.90 | 0.47 |
| 16 | DeKalb, IL | 03/05/2007 | 16/07/2007 | Poncho 1250 | 48 | 1.25 | 0.6 | Clothianidin | s | 2.18 | 79 | 1.18 | 0.84 | 1.11 | 0.58 |
| 17 | DeKalb, IL | 03/05/2007 | 16/07/2007 | Force 2.25CS | 23.4 | 0.46 | 10.01 | Tefluthrin | b | 2.18 | 79 | 0.45 | 0.72 | 0.94 | 0.49 |
| 18 | DeKalb, IL | 03/05/2007 | 16/07/2007 | Force 3G | 3 | 4 | 11.16 | Tefluthrin | b | 2.18 | 79 | 0.74 | 0.63 | 0.82 | 0.43 |
| 19 | DeKalb, IL | 03/05/2007 | 07/08/2007 | Poncho 1250 | 48 | 1.25 | 0.6 | Clothianidin | s | 2.49 | 79 | 1.21 | 0.95 | 1.25 | 0.66 |
| 20 | DeKalb, IL | 05/05/2008 | 29/07/2008 | Lorsban 15G | 15 | 8 | 111.61 | Chlorpyrifos | b | 2.88 | 200 | 1.55 | 1.93 | 2.52 | 1.33 |
| 21 | DeKalb, IL | 05/05/2008 | 29/07/2008 | Poncho 1250 | 48 | 1.25 | 0.6 | Clothianidin | s | 2.88 | 200 | 2.85 | 2.66 | 3.00 | 1.84 |
| No. | Location  ^*^ | Planting date  ^*^ | Assessment date  ^*^ | Treatment  ^*^ | % active ingredient in product | Pesticide application (oz/1000 ft of row for band and furrow application; mg/seed for seed application) ^*^ | Application rate (mg a.s. /m row) or mg/seed for seed treatment | Active substance | Placement type  ^*^ | Injury value observed in the control  ^*^ | Fitted pest pressure for the control | Injury value observed in the treatment^*^ | Injury value predicted average | Injury value predicted upper 95% CI | Injury value predicted lower 95% CI |
| 22 | DeKalb, IL | 05/05/2008 | 29/07/2008 | Force 2.25CS | 23.4 | 0.46 | 10.01 | Tefluthrin | b | 2.88 | 200 | 1.06 | 2.12 | 2.78 | 1.47 |
| 23 | DeKalb, IL | 05/05/2008 | 11/08/2008 | Poncho 1250 | 48 | 1.25 | 0.6 | Clothianidin | s | 2.88 | 200 | 2.64 | 2.58 | 3.00 | 1.78 |
| 24 | DeKalb, IL | 05/05/2008 | 11/08/2008 | Force 2.25CS | 23.4 | 0.46 | 10.01 | Tefluthrin | b | 2.88 | 200 | 1.10 | 2.07 | 2.72 | 1.43 |
| 25 | DeKalb, IL | 24/05/2009 | 29/07/2009 | Lorsban 15G | 15 | 8 | 111.61 | Chlorpyrifos | b | 0.78 | 64 | 0.38 | 0.26 | 0.34 | 0.18 |
| 26 | DeKalb, IL | 24/05/2009 | 29/07/2009 | Force 2.1CS | 23.4 | 0.46 | 10.01 | Tefluthrin | b | 0.78 | 64 | 0.23 | 0.29 | 0.38 | 0.20 |
| 27 | DeKalb, IL | 10/05/2010 | 14/07/2010 | Force 2.1CS | 23.4 | 0.46 | 10.01 | Tefluthrin | b | 0.63 | 75 | 0.09 | 0.25 | 0.33 | 0.17 |
| 28 | DeKalb, IL | 10/05/2011 | 18/07/2011 | Poncho 1250 | 48 | 1.25 | 0.6 | Clothianidin | s | 0.98 | 43 | 0.13 | 0.32 | 0.42 | 0.22 |
| 29 | DeKalb, IL | 23/04/2012 | 16/07/2012 | Force CS | 23.4 | 0.46 | 10.01 | Tefluthrin | b | 1.69 | 98 | 0.81 | 0.42 | 0.56 | 0.29 |
| 30 | DeKalb, IL | 14/05/2013 | 30/07/2013 | Force CS | 23.4 | 0.46 | 10.01 | Tefluthrin | b | 0.18 | 15 | 0.08 | 0.15 | 0.19 | 0.10 |
| 31 | DeKalb, IL | 08/05/2014 | 28/07/2014 | Belay liquid | 23 | 0.36 | 7.7 | Clothianidin | f | 2.10 | 75 | 0.10 | 0.69 | 0.90 | 0.47 |
| 32 | DeKalb, IL | 08/05/2014 | 28/07/2014 | Belay liquid | 23 | 0.56 | 11.98 | Clothianidin | f | 2.10 | 75 | 0.02 | 0.72 | 0.94 | 0.49 |
| 33 | DeKalb, IL | 08/05/2014 | 28/07/2014 | Belay liquid | 23 | 0.62 | 13.26 | Clothianidin | f | 2.10 | 75 | 0.29 | 0.77 | 1.01 | 0.53 |
| 34 | DeKalb, IL | 08/05/2014 | 28/07/2014 | Force CS | 23.4 | 0.46 | 10.01 | Tefluthrin | f | 1.83 | 75 | 0.05 | 0.68 | 0.88 | 0.47 |
| 35 | Monmouth, IL | 28/04/2005 | 25/07/2005 | Lorsban 15G | 15 | 8 | 111.61 | Chlorpyrifos | b | 2.25 | 151 | 0.46 | 0.66 | 0.86 | 0.45 |
| 36 | Monmouth, IL | 28/04/2005 | 25/07/2005 | Lorsban 4E | 49.9 | 2.4 | 111.39 | Chlorpyrifos | b | 2.25 | 151 | 0.26 | 0.67 | 0.87 | 0.46 |
| 37 | Monmouth, IL | 28/04/2005 | 25/07/2005 | Lorsban 75 WG | 75 | 1.2 | 83.71 | Chlorpyrifos | b | 2.25 | 151 | 0.48 | 0.68 | 0.89 | 0.47 |
| 38 | Monmouth, IL | 28/04/2005 | 25/07/2005 | Nufos 15G | 15 | 8 | 111.61 | Chlorpyrifos | b | 2.25 | 151 | 0.35 | 0.69 | 0.90 | 0.47 |
| 39 | Monmouth, IL | 28/04/2005 | 25/07/2005 | Poncho 1250 | 48 | 1.25 | 0.6 | Clothianidin | s | 2.25 | 151 | 0.55 | 1.03 | 1.35 | 0.71 |
| 40 | Monmouth, IL | 28/04/2005 | 25/07/2005 | Force 3G | 3 | 4 | 11.16 | Tefluthrin | b | 2.25 | 151 | 0.41 | 0.76 | 1.00 | 0.53 |
| 41 | Monmouth, IL | 28/04/2005 | 25/07/2005 | Force 3G, modified SmartBox metering units | 3 | 4 | 11.16 | Tefluthrin | b | 2.25 | 151 | 0.41 | 0.73 | 0.96 | 0.51 |
| 42 | Monmouth, IL | 04/05/2006 | 24/07/2006 | Lorsban 15G | 15 | 8 | 111.61 | Chlorpyrifos | b | 2.98 | 228 | 0.47 | 1.32 | 1.73 | 0.91 |
| 43 | Monmouth, IL | 04/05/2006 | 24/07/2006 | Poncho 1250 | 48 | 1.25 | 0.6 | Clothianidin | s | 2.98 | 228 | 1.65 | 2.15 | 2.81 | 1.48 |
| 44 | Monmouth, IL | 04/05/2006 | 24/07/2006 | Force 3G | 3 | 4 | 11.16 | Tefluthrin | b | 2.98 | 228 | 0.57 | 1.54 | 2.01 | 1.06 |
| 45 | Monmouth, IL | 04/05/2006 | 08/08/2006 | Poncho 1250 | 48 | 1.25 | 0.6 | Clothianidin | s | 2.82 | 228 | 1.72 | 2.25 | 2.95 | 1.56 |
| 46 | Monmouth, IL | 10/05/2007 | 12/07/2007 | Lorsban 15G | 15 | 8 | 111.61 | Chlorpyrifos | b | 1.14 | 190 | 0.20 | 0.50 | 0.66 | 0.35 |
| No. | Location  ^*^ | Planting date  ^*^ | Assessment date  ^*^ | Treatment  ^*^ | % active ingredient in product | Pesticide application (oz/1000 ft of row for band and furrow application; mg/seed for seed application) ^*^ | Application rate (mg a.s. /m row) or mg/seed for seed treatment | Active substance | Placement type  ^*^ | Injury value observed in the control  ^*^ | Fitted pest pressure for the control | Injury value observed in the treatment^*^ | Injury value predicted average | Injury value predicted upper 95% CI | Injury value predicted lower 95% CI |
| 47 | Monmouth, IL | 10/05/2007 | 12/07/2007 | Lorsban 4E | 49.9 | 2.4 | 111.39 | Chlorpyrifos | b | 1.14 | 190 | 0.25 | 0.54 | 0.71 | 0.38 |
| 48 | Monmouth, IL | 10/05/2007 | 12/07/2007 | Poncho 1250 | 48 | 1.25 | 0.6 | Clothianidin | s | 1.14 | 190 | 0.90 | 0.68 | 0.89 | 0.47 |
| 49 | Monmouth, IL | 10/05/2007 | 12/07/2007 | Force 2.25CS | 23.4 | 0.46 | 10.01 | Tefluthrin | b | 1.14 | 190 | 0.23 | 0.56 | 0.73 | 0.39 |
| 50 | Monmouth, IL | 10/05/2007 | 12/07/2007 | Force 3G | 3 | 4 | 11.16 | Tefluthrin | b | 1.14 | 190 | 0.22 | 0.54 | 0.71 | 0.37 |
| 51 | Monmouth, IL | 10/05/2007 | 06/08/2007 | Poncho 1250 | 48 | 1.25 | 0.6 | Clothianidin | s | 1.20 | 190 | 0.55 | 0.72 | 0.94 | 0.50 |
| 52 | Monmouth, IL | 23/04/2008 | 16/07/2008 | Lorsban 15G | 15 | 8 | 111.61 | Chlorpyrifos | b | 1.09 | 250 | 0.85 | 0.99 | 1.29 | 0.68 |
| 53 | Monmouth, IL | 23/04/2008 | 16/07/2008 | Poncho 1250 | 48 | 1.25 | 0.6 | Clothianidin | s | 1.09 | 250 | 1.21 | 1.13 | 1.48 | 0.78 |
| 54 | Monmouth, IL | 23/04/2008 | 16/07/2008 | Force 2.25CS | 23.4 | 0.46 | 10.01 | Tefluthrin | b | 1.09 | 250 | 0.62 | 1.28 | 1.67 | 0.88 |
| 55 | Monmouth, IL | 23/04/2008 | 16/07/2008 | Force 3G | 3 | 4 | 11.16 | Tefluthrin | b | 1.09 | 250 | 0.13 | 1.24 | 1.63 | 0.86 |
| 56 | Monmouth, IL | 23/04/2008 | 12/08/2008 | Poncho 1250 | 48 | 1.25 | 0.6 | Clothianidin | s | 1.38 | 250 | 1.83 | 1.08 | 1.41 | 0.74 |
| 57 | Monmouth, IL | 23/04/2008 | 12/08/2008 | Force 2.25CS | 23.4 | 0.46 | 10.01 | Tefluthrin | b | 1.38 | 250 | 0.78 | 1.23 | 1.61 | 0.85 |
| 58 | Monmouth, IL | 05/05/2009 | 20/07/2009 | Lorsban 15G | 15 | 8 | 111.61 | Chlorpyrifos | b | 0.90 | 46 | 0.28 | 0.29 | 0.38 | 0.20 |
| 59 | Monmouth, IL | 05/05/2009 | 20/07/2009 | Force 2.1CS | 23.4 | 0.46 | 10.01 | Tefluthrin | b | 0.90 | 46 | 0.08 | 0.28 | 0.36 | 0.19 |
| 60 | Monmouth, IL | 02/05/2011 | 12/07/2011 | Poncho 1250 | 48 | 1.25 | 0.6 | Clothianidin | s | 0.11 | 12 | 0.11 | 0.08 | 0.11 | 0.06 |
| 61 | Monmouth, IL | 21/04/2012 | 10/07/2012 | Force CS | 23.4 | 0.46 | 10.01 | Tefluthrin | b | 0.10 | 17 | 0.06 | 0.14 | 0.18 | 0.09 |
| 62 | Monmouth, IL | 01/05/2013 | 17/07/2013 | Belay liquid | 23 | 0.62 | 13.26 | Clothianidin | f | 0.69 | 41 | 0.27 | 0.30 | 0.40 | 0.21 |
| 63 | Monmouth, IL | 01/05/2013 | 17/07/2013 | Force CS | 23.4 | 0.46 | 10.01 | Tefluthrin | b | 0.69 | 41 | 0.14 | 0.31 | 0.40 | 0.21 |
| 64 | Monmouth, IL | 07/05/2014 | 14/07/2014 | Force CS | 23.4 | 0.46 | 10.01 | Tefluthrin | f | 2.18 | 111 | 0.57 | 0.69 | 0.90 | 0.48 |
| 65 | Perry, IL | 24/04/2006 | 18/07/2006 | Lorsban 15G | 15 | 8 | 111.61 | Chlorpyrifos | b | 0.41 | 28 | 0.11 | 0.29 | 0.39 | 0.20 |
| 66 | Perry, IL | 24/04/2006 | 18/07/2006 | Poncho 1250 | 48 | 1.25 | 0.6 | Clothianidin | s | 0.41 | 28 | 0.09 | 0.33 | 0.44 | 0.23 |
| 67 | Perry, IL | 24/04/2006 | 18/07/2006 | Force 3G | 3 | 4 | 11.16 | Tefluthrin | b | 0.41 | 28 | 0.31 | 0.32 | 0.42 | 0.22 |
| 68 | Perry, IL | 08/05/2007 | 09/07/2007 | Lorsban 15G | 15 | 8 | 111.61 | Chlorpyrifos | b | 0.99 | 182 | 0.10 | 0.53 | 0.70 | 0.37 |
| 69 | Perry, IL | 08/05/2007 | 09/07/2007 | Lorsban 4E | 49.9 | 2.4 | 111.39 | Chlorpyrifos | b | 0.99 | 182 | 0.08 | 0.52 | 0.68 | 0.36 |
| 70 | Perry, IL | 08/05/2007 | 09/07/2007 | Poncho 1250 | 48 | 1.25 | 0.6 | Clothianidin | s | 0.99 | 182 | 0.15 | 0.55 | 0.73 | 0.38 |
| 71 | Perry, IL | 08/05/2007 | 09/07/2007 | Force 2.25CS | 23.4 | 0.46 | 10.01 | Tefluthrin | b | 0.99 | 182 | 0.12 | 0.50 | 0.66 | 0.35 |
| 72 | Perry, IL | 08/05/2007 | 09/07/2007 | Force 3G | 3 | 4 | 11.16 | Tefluthrin | b | 0.99 | 182 | 0.10 | 0.57 | 0.75 | 0.39 |
| 73 | Perry, IL | 30/04/2008 | 16/07/2008 | Lorsban 15G | 15 | 8 | 111.61 | Chlorpyrifos | b | 0.69 | 62 | 1.52 | 0.46 | 0.61 | 0.32 |
| No. | Location  ^*^ | Planting date  ^*^ | Assessment date  ^*^ | Treatment  ^*^ | % active ingredient in product | Pesticide application (oz/1000 ft of row for band and furrow application; mg/seed for seed application) ^*^ | Application rate (mg a.s. /m row) or mg/seed for seed treatment | Active substance | Placement type  ^*^ | Injury value observed in the control  ^*^ | Fitted pest pressure for the control | Injury value observed in the treatment^*^ | Injury value predicted average | Injury value predicted upper 95% CI | Injury value predicted lower 95% CI |
| 74 | Perry, IL | 30/04/2008 | 16/07/2008 | Poncho 1250 | 48 | 1.25 | 0.6 | Clothianidin | s | 0.69 | 40 | 0.74 | 0.35 | 0.46 | 0.24 |
| 75 | Perry, IL | 30/04/2008 | 16/07/2008 | Force 2.25CS | 23.4 | 0.46 | 10.01 | Tefluthrin | b | 0.69 | 40 | 0.22 | 0.27 | 0.36 | 0.19 |
| 76 | Perry, IL | 30/04/2008 | 12/08/2008 | Poncho 1250 | 48 | 1.25 | 0.6 | Clothianidin | s | 0.71 | 40 | 0.75 | 0.40 | 0.53 | 0.28 |
| 77 | Perry, IL | 30/04/2008 | 12/08/2008 | Force 2.25CS | 23.4 | 0.46 | 10.01 | Tefluthrin | b | 0.71 | 40 | 0.20 | 0.37 | 0.48 | 0.25 |
| 78 | Perry, IL | 23/04/2009 | 20/07/2009 | Lorsban 15G | 15 | 8 | 111.61 | Chlorpyrifos | b | 0.20 | 18 | 0.18 | 0.26 | 0.34 | 0.18 |
| 79 | Perry, IL | 23/04/2009 | 20/07/2009 | Force 2.1CS | 23.4 | 0.46 | 10.01 | Tefluthrin | b | 0.20 | 18 | 0.03 | 0.26 | 0.34 | 0.18 |
| 80 | Perry, IL | 03/05/2011 | 12/07/2011 | Poncho 1250 | 48 | 1.25 | 0.6 | Clothianidin | s | 0.21 | 39 | 0.09 | 0.14 | 0.18 | 0.09 |
| 81 | Perry, IL | 19/04/2012 | 10/07/2012 | Force CS | 23.4 | 0.46 | 10.01 | Tefluthrin | b | 1.40 | 110 | 0.28 | 0.37 | 0.48 | 0.25 |
| 82 | Perry, IL | 01/05/2013 | 29/07/2013 | Force CS | 23.4 | 0.46 | 10.01 | Tefluthrin | b | 0.69 | 35 | 0.08 | 0.30 | 0.39 | 0.21 |
| 83 | Urbana, IL | 03/05/2005 | 13/07/2005 | Lorsban 15G | 15 | 8 | 111.61 | Chlorpyrifos | b | 2.32 | 161 | 0.66 | 0.68 | 0.89 | 0.47 |
| 84 | Urbana, IL | 03/05/2005 | 13/07/2005 | Lorsban 4E | 49.9 | 2.4 | 111.39 | Chlorpyrifos | b | 2.32 | 161 | 0.91 | 0.68 | 0.89 | 0.47 |
| 85 | Urbana, IL | 03/05/2005 | 13/07/2005 | Lorsban 75 WG | 75 | 1.2 | 83.71 | Chlorpyrifos | b | 2.32 | 161 | 1.33 | 0.73 | 0.96 | 0.50 |
| 86 | Urbana, IL | 03/05/2005 | 13/07/2005 | Nufos 15G | 15 | 8 | 111.61 | Chlorpyrifos | b | 2.32 | 161 | 0.53 | 0.71 | 0.93 | 0.49 |
| 87 | Urbana, IL | 03/05/2005 | 13/07/2005 | Poncho 1250 | 48 | 1.25 | 0.6 | Clothianidin | s | 2.32 | 161 | 1.21 | 1.00 | 1.31 | 0.69 |
| 88 | Urbana, IL | 03/05/2005 | 13/07/2005 | Force 3G | 3 | 4 | 11.16 | Tefluthrin | b | 2.32 | 161 | 0.48 | 0.79 | 1.04 | 0.55 |
| 89 | Urbana, IL | 03/05/2005 | 13/07/2005 | Force 3G, modified SmartBox metering units | 3 | 4 | 11.16 | Tefluthrin | b | 2.32 | 161 | 0.52 | 0.83 | 1.09 | 0.57 |
| 90 | Urbana, IL | 28/04/2006 | 17/07/2006 | Lorsban 15G | 15 | 8 | 111.61 | Chlorpyrifos | b | 2.95 | 159 | 0.63 | 1.27 | 1.66 | 0.88 |
| 91 | Urbana, IL | 28/04/2006 | 17/07/2006 | Poncho 1250 | 48 | 1.25 | 0.6 | Clothianidin | s | 2.95 | 159 | 1.97 | 2.12 | 2.78 | 1.46 |
| 92 | Urbana, IL | 28/04/2006 | 17/07/2006 | Force 3G | 3 | 4 | 11.16 | Tefluthrin | b | 2.95 | 159 | 1.01 | 1.38 | 1.80 | 0.95 |
| 93 | Urbana, IL | 28/04/2006 | 07/08/2006 | Poncho 1250 | 48 | 1.25 | 0.6 | Clothianidin | s | 3.00 | 159 | 2.35 | 2.21 | 2.89 | 1.52 |
| 94 | Urbana, IL | 01/05/2007 | 09/07/2007 | Lorsban 15G | 15 | 8 | 111.61 | Chlorpyrifos | b | 2.74 | 373 | 0.40 | 1.08 | 1.41 | 0.74 |
| 95 | Urbana, IL | 01/05/2007 | 09/07/2007 | Lorsban 4E | 49.9 | 2.4 | 111.39 | Chlorpyrifos | b | 2.74 | 373 | 0.34 | 1.18 | 1.55 | 0.81 |
| 96 | Urbana, IL | 01/05/2007 | 09/07/2007 | Saurus 15G | 15 | 8 | 111.61 | Chlorpyrifos | b | 2.74 | 373 | 0.26 | 1.04 | 1.36 | 0.72 |
| 97 | Urbana, IL | 01/05/2007 | 09/07/2007 | Saurus 15G | 15 | 8 | 111.61 | Chlorpyrifos | f | 2.74 | 373 | 0.31 | 1.24 | 1.62 | 0.85 |
| 98 | Urbana, IL | 01/05/2007 | 09/07/2007 | Poncho 1250 | 48 | 1.25 | 0.6 | Clothianidin | s | 2.74 | 373 | 1.49 | 1.50 | 1.96 | 1.03 |
| No. | Location  ^*^ | Planting date  ^*^ | Assessment date  ^*^ | Treatment  ^*^ | % active ingredient in product | Pesticide application (oz/1000 ft of row for band and furrow application; mg/seed for seed application) ^*^ | Application rate (mg a.s. /m row) or mg/seed for seed treatment | Active substance | Placement type  ^*^ | Injury value observed in the control  ^*^ | Fitted pest pressure for the control | Injury value observed in the treatment^*^ | Injury value predicted average | Injury value predicted upper 95% CI | Injury value predicted lower 95% CI |
| 99 | Urbana, IL | 01/05/2007 | 09/07/2007 | Force 2.25CS | 23.4 | 0.46 | 10.01 | Tefluthrin | b | 2.74 | 373 | 0.36 | 1.15 | 1.50 | 0.79 |
| 100 | Urbana, IL | 01/05/2007 | 09/07/2007 | Force 3G | 3 | 4 | 11.16 | Tefluthrin | b | 2.74 | 373 | 0.41 | 1.14 | 1.49 | 0.79 |
| 101 | Urbana, IL | 01/05/2007 | 07/08/2007 | Poncho 1250 | 48 | 1.25 | 0.6 | Clothianidin | s | 2.66 | 373 | 1.21 | 1.42 | 1.86 | 0.98 |
| 102 | Urbana, IL | 07/05/2007 | 09/07/2007 | Force 2.25CS | 23.4 | 0.12 | 2.61 | Tefluthrin | b | 1.38 | 223 | 0.14 | 0.66 | 0.86 | 0.45 |
| 103 | Urbana, IL | 07/05/2007 | 09/07/2007 | Force 2.25CS | 23.4 | 0.12 | 2.61 | Tefluthrin | f | 1.38 | 223 | 0.13 | 0.61 | 0.79 | 0.42 |
| 104 | Urbana, IL | 07/05/2007 | 09/07/2007 | Force 3G | 3 | 4 | 11.16 | Tefluthrin | b | 1.38 | 223 | 0.16 | 0.56 | 0.73 | 0.38 |
| 105 | Urbana, IL | 07/05/2007 | 09/07/2007 | Force 3G | 3 | 4 | 11.16 | Tefluthrin | f | 1.38 | 223 | 0.08 | 0.61 | 0.80 | 0.42 |
| 106 | Urbana, IL | 24/04/2008 | 22/07/2008 | Lorsban 15G | 15 | 8 | 111.61 | Chlorpyrifos | b | 1.75 | 213 | 0.66 | 1.43 | 1.88 | 0.99 |
| 107 | Urbana, IL | 24/04/2008 | 22/07/2008 | Poncho 1250 | 48 | 1.25 | 0.6 | Clothianidin | s | 1.75 | 213 | 2.07 | 1.40 | 1.83 | 0.96 |
| 108 | Urbana, IL | 24/04/2008 | 22/07/2008 | Force 2.25CS | 23.4 | 0.46 | 10.01 | Tefluthrin | b | 1.75 | 213 | 0.39 | 1.55 | 2.03 | 1.07 |
| 109 | Urbana, IL | 24/04/2008 | 22/07/2008 | Force 3G | 3 | 4 | 11.16 | Tefluthrin | b | 1.75 | 213 | 0.21 | 1.28 | 1.68 | 0.89 |
| 110 | Urbana, IL | 24/04/2008 | 12/08/2008 | Poncho 1250 | 48 | 1.25 | 0.6 | Clothianidin | s | 1.99 | 213 | 2.18 | 1.54 | 2.02 | 1.06 |
| 111 | Urbana, IL | 24/04/2008 | 12/08/2008 | Force 2.25CS | 23.4 | 0.46 | 10.01 | Tefluthrin | b | 1.99 | 213 | 0.16 | 1.59 | 2.08 | 1.10 |
| 112 | Urbana, IL | 18/04/2009 | 22/07/2009 | Lorsban 15G | 15 | 8 | 111.61 | Chlorpyrifos | b | 2.16 | 83 | 0.05 | 0.72 | 0.94 | 0.50 |
| 113 | Urbana, IL | 18/04/2009 | 22/07/2009 | Force 2.1CS | 23.4 | 0.46 | 10.01 | Tefluthrin | b | 2.16 | 83 | 0.18 | 0.66 | 0.86 | 0.45 |
| 114 | Urbana, IL | 04/05/2010 | 12/07/2010 | Force 2.1CS | 23.4 | 0.46 | 10.01 | Tefluthrin | b | 0.56 | 61 | 0.13 | 0.23 | 0.30 | 0.16 |
| 115 | Urbana, IL | 11/05/2011 | 11/07/2011 | Poncho 1250 | 48 | 1.25 | 0.6 | Clothianidin | s | 0.87 | 91 | 0.68 | 0.40 | 0.53 | 0.28 |
| 116 | Urbana, IL | 18/04/2012 | 09/07/2012 | Force CS | 23.4 | 0.46 | 10.01 | Tefluthrin | b | 1.94 | 100 | 0.98 | 0.41 | 0.53 | 0.28 |
| 117 | Urbana, IL | 16/05/2013 | 18/07/2013 | Belay liquid | 23 | 0.62 | 13.26 | Clothianidin | f | 1.34 | 171 | 0.25 | 0.48 | 0.63 | 0.33 |
| 118 | Urbana, IL | 16/05/2013 | 18/07/2013 | Force CS | 23.4 | 0.46 | 10.01 | Tefluthrin | b | 1.34 | 171 | 0.15 | 0.55 | 0.72 | 0.38 |
| 119 | Urbana, IL | 12/05/2014 | 23/07/2014 | Belay liquid | 23 | 0.36 | 7.7 | Clothianidin | f | 1.40 | 108 | 0.23 | 0.50 | 0.66 | 0.35 |
| 120 | Urbana, IL | 12/05/2014 | 23/07/2014 | Belay liquid | 23 | 0.56 | 11.98 | Clothianidin | f | 1.40 | 108 | 0.20 | 0.43 | 0.57 | 0.30 |
| 121 | Urbana, IL | 12/05/2014 | 23/07/2014 | Belay liquid | 23 | 0.62 | 13.26 | Clothianidin | f | 1.40 | 108 | 0.30 | 0.57 | 0.75 | 0.39 |
| 122 | Urbana, IL | 12/05/2014 | 23/07/2014 | Force CS | 23.4 | 0.46 | 10.01 | Tefluthrin | f | 1.40 | 108 | 0.36 | 0.43 | 0.56 | 0.30 |

s= seed; f = furrow; b = band; * = data from the literature [1]

Damage assessment in 13 trials was undertaken twice (early and late assessment); these assessments were treated as separate field trials for model evaluation.

Table S3: Survival of western corn rootworm larvae (second instar) in toxicity tests with chlorpyrifos, clothianidin, and tefluthrin.

| **Chlorpyrifos** | | | | | | |
| --- | --- | --- | --- | --- | --- | --- |
| Concentration [mg/L] | 0 | 0.05 | 0.15 | 0.44 | 1.33 | 4 |
| Time (d) | # alive | # alive | # alive | # alive | # alive | # alive |
| 0 | 30 | 30 | 30 | 30 | 30 | 30 |
| 1 | 25 | 26 | 26 | 25 | 1 | 0 |
| 2 | 25 | 21 | 16 | 16 | 0 | 0 |
| 5 | 26 | 24 | 18 | 12 | 0 | 0 |
| **Clothianidin** | | | | | | |
| Concentration [mg/L] | 0 | 0.05 | 0.15 | 0.44 | 1.33 | 4 |
| Time (d) | # alive | # alive | # alive | # alive | # alive | # alive |
| 0 | 30 | 30 | 30 | 30 | 30 | 30 |
| 1 | 26 | 23 | 19 | 11 | 7 | 1 |
| 2 | 29 | 23 | 5 | 0 | 1 | 0 |
| 5 | 28 | 22 | 6 | 0 | 0 | 0 |
| **Tefluthrin** | | | | | | |
| Concentration [mg/L] | 0 | 0.05 | 0.15 | 0.44 | 1.33 | 4 |
| Time (d) | # alive | # alive | # alive | # alive | # alive | # alive |
| 0 | 30 | 30 | 30 | 30 | 30 | 30 |
| 1 | 30 | 27 | 28 | 24 | 0 | 0 |
| 2 | 28 | 24 | 21 | 5 | 1 | 0 |
| 5 | 29 | 16 | 12 | 3 | 0 | 0 |

Table S4: Soil characteristics of the four field trial sites used for model parameterization.

|  | Horizon | Depth | Total porosity | Field capacity | Wilting point | Bulk density | Organic carbon |
| --- | --- | --- | --- | --- | --- | --- | --- |
|  |  | (cm) | (cm^3^/cm^3^) | (cm^3^/cm^3^) | (cm^3^/cm^3^) | (g/cm^3^) | (%) |
| DeKalb  (Flanagan series) | A | 0-46 | 0.48 | 0.42 | 0.19 | 1.3 | 2.4 |
|  | B | 46-96 | 0.48 | 0.43 | 0.23 | 1.35 | 0.6 |
|  | C | 96-100 | 0.48 | 0.43 | 0.23 | 1.35 | 0.6 |
| Urbana  (Drummer series) | A | 0-36 | 0.51 | 0.44 | 0.22 | 1.2 | 3.2 |
|  | B | 360-100 | 0.48 | 0.43 | 0.2 | 1.32 | 0.7 |
| Perry  (Downsouth series) | A | 0-28 | 0.49 | 0.42 | 0.19 | 1.25 | 1.8 |
|  | B | 28-100 | 0.48 | 0.4 | 0.2 | 1.35 | 0.6 |
| Monmouth (Muscatane series) | A | 0-40 | 0.46 | 0.41 | 0.18 | 1.35 | 2.5 |
|  | B | 40-56 | 0.46 | 0.41 | 0.21 | 1.4 | 0.6 |
|  | C | 56-100 | 0.44 | 0.39 | 0.2 | 1.45 | 0.6 |

Table S5: Properties of the pesticides [3].

|  | **Tefluthrin** | **Clothianidin** | **Chlorpyrifos** |
| --- | --- | --- | --- |
| Substance group | Pyrethroid | Neonicotinoid | Organophosphate |
| Mode of action | Sodium channel modulator | Acetylcholine receptor (nAChR) agonist | Acetylcholinesterase (AChE) inhibitor |
|  | Contact and respiratory action with some repellent effects | Translaminar and root systemic activity | Nonsystemic with contact and stomach action |
| Molecular mass [g/mol] | 418.73 | 249.7 | 350.89 |
| Vapor pressure (mPa) | 8.4 | 2.8 × 10^-8^ | 1.43 |
| Half-life in soil at 20°C [d] | 37 | 545 | 76 |
| Soil organic carbon partition coefficient [L/kg] | 112900 | 123 | 8151 |
| Water solubility [mg/L] | 0.016 | 340 | 1.5 |

**Details on the cost-benefit analysis:**

The economic threshold triggering pesticide application is defined as the level of root damage at which yield loss can be expected (i.e., 0.25 NIS for rootworm in the US [4]). The economic injury level is the point at which control delivers sufficient additional yield (i.e., revenue) to cover the treatment costs. The calculation of the change in revenue for pesticide treatment at different application rates used the scenario-specific NIS and the average and standard deviation of the conversion of NIS to yield loss (16.5±1.9%/NIS value) calculated from the literature [5,6]. The calculation assumed a maximum yield without any pest damage of 12.5 tons/ha (200 bushels/acre), a price for corn of $165/ton (representing the average price for corn of $4.2/bushel from 2014 for Central Illinois), and an insecticide cost of $0.55/ha for each g a.s./ha. The latter was derived using an insecticide cost of $36/ha, which was taken as the average from an evaluation of economic damage for neonicotinoid seed treatments [7] and equating this cost to an application rate of 65 g a.s./ha, which achieved 95% of the maximum achievable efficacy. The change in revenue was calculated as the difference between the additional income achieved from the yield loss avoided through reduced pest damage and the cost of the pesticide treatment.

**References:**

1. University of Illinois Extension, 2005-2014. Summary of Field Crop Insect Management Trials. *On Target*, College of Agricultural, Consumer and Environmental Sciences. Department of Crop Sciences, IL, USA. <https://ipm.illinois.edu/ontarget/pastissues.html>. Last accessed 10.11.2018.
2. Oleson, J.D., Park, Y-L., Nowatzki, T.M., Tollefson, J.J., 2005. Node-injury scale to evaluate root injury by corn rootworms (Coleoptera: Chrysomelidae) J. Econ. Entomol. 98, 1-8.
3. University of Hertfordshire, 2013. The Pesticide Properties DataBase (PPDB) developed by the Agriculture & Environment Research Unit (AERU), https://sitem.herts.ac.uk/aeru/ppdb/en/atoz.htm Last accessed 10.11.2018.
4. Hodgson, E., Gassmann, A., 2013. Time to assess corn rootworm activity in Iowa. <http://www.extension.iastate.edu/CropNews/2013/0731hodgsongassman.htm>. Last accessed 10.11.2018.
5. Dun, Z., Mitchell, P.D., Agosti, M., 2010. Estimating *Diabrotica virgifera virgifera* damage functions with field trial data: applying an unbalanced nested error component model. J. Appl. Entomol. 134, 409-419.
6. Tinsley, N.A., Estes R.E., Gray, M.E., 2013. Validation of a nested error component model to estimate damage caused by corn rootworm larvae. J. of Appl. Entomol. 137, 161-169.
7. Alford, A., Krupke, C.H., 2017. Translocation of the neonicotinoid seed treatment clothianidin in maize. PLOS ONE 12, e0173836.

1. Current address: ibacon GmbH, Arheilger Weg 17, D-64380 Rossdorf, Germany [↑](#footnote-ref-1)
2. Current address: Syngenta Crop Protection AG, Schwarzwaldallee 215, Basel, CH-4002, Switzerland [↑](#footnote-ref-2)
